# Residue-Specific Structural and Dynamical Coupling of Protein and Hydration Water Revealed by Molecular Dynamics Simulations

**DOI:** 10.3390/biom15050660

**Published:** 2025-05-02

**Authors:** Shuai Wang, Jun Gao, Xiakun Chu

**Affiliations:** 1 College of Informatics, Huazhong Agricultural University, Wuhan 430070, China; shuaiwang@hkust-gz.edu.cn (S.W.); gaojun@mail.hzau.edu.cn (J.G.); 2 Advanced Materials Thrust, Function Hub, The Hong Kong University of Science and Technology (Guangzhou), Guangzhou 511400, China; 3 Guangzhou Municipal Key Laboratory of Materials Informatics, The Hong Kong University of Science and Technology (Guangzhou), Guangzhou 511400, China; 4 Division of Life Science, The Hong Kong University of Science and Technology, Clear Water Bay, Hong Kong SAR 999077, China

**Keywords:** autocorrelation function, relaxation time, protein–water interactions, protein-water coupling, translational order, structural order, orientational order, tetrahedral order

## Abstract

Proteins and their surrounding hydration water engage in a dynamic interplay that is critical for maintaining structural stability and functional integrity. However, the intricate coupling between protein dynamics and the structural order of hydration water remains poorly understood. Here, we employ all-atom molecular dynamics simulations to investigate this relationship across four representative proteins. Our results reveal that protein residues with greater flexibility or solvent exposure are surrounded by more disordered hydration water, akin to bulk water, whereas rigid and buried non-polar residues are associated with structurally ordered hydration shells. Due to their strong hydrogen bonding and electrostatic interactions, charged residues exhibit the most disordered hydration water, while non-polar residues are associated with the structurally most ordered hydration water. We further uncovered a positive correlation between the relaxation dynamics of protein residues and their hydration water: slower (faster) protein relaxation is coupled with slower (faster) relaxation of the structural order of hydration water. Notably, this coupling weakens with increasing residue flexibility or solvent exposure, with non-polar residues displaying the strongest coupling, and charged residues the weakest. To further uncover their coupling mechanism, we elucidate residue-specific coupled fluctuations between protein residues and hydration water by generating scatter plots. These findings provide a comprehensive understanding of the mechanisms underlying protein–water interactions, offering valuable insights into the role of hydration water in protein stability, dynamics, and function.

## 1. Introduction

Proteins operate in aqueous environments, where hydration water near their surface interacts dynamically with the protein [1]. Increasing evidence suggests that hydration water is far from being an inert medium. Instead, it plays a critical role in regulating protein folding and structural stability [2,3,4,5], protein dynamics [2,3,6,7], and overall protein function [2,3,6]. At the same time, the presence of a solute, such as a protein, significantly alters the thermodynamic and dynamic properties of the surrounding hydration water [8,9,10,11,12]. As a result, proteins and hydration water mutually influence one another, leading to coupled thermodynamic and dynamic behaviors [2,7,13,14]. However, the detailed mechanisms underlying this coupling remain poorly understood, limiting our ability to fully comprehend how hydration water modulates protein behavior.

Current research has focused on how proteins perturb the structural properties of hydration water, including the thickness, mass density, and hydrogen bond network of the hydration shell [2,8,12,15]. The local structural order of water molecules is known to play a critical role in their thermodynamic and dynamic anomalous behaviors [16,17,18,19]. While traditional order parameters capture certain aspects of water structure, they often fail to fully characterize the structural properties of hydration water at the protein–water interface [20,21,22,23,24,25,26,27,28,29,30,31]. One of the most widely used tetrahedral order parameters, q, characterizing the rotational (tetrahedral) symmetry is determined only by the angular distribution of neighbors in the first shell. However, the rotational symmetry of the interfacial water will inevitably be disrupted in the presence of protein, making many interfacial water molecules have less than four neighbors in the first coordination shell, leading to inaccurate calculation of the ordering of the interfacial water structure [22]. Filtering out water molecules with less than four neighbors is a method to evaluate the tetrahedral order parameter [32] but it could impact the statistics of interfacial water. Furthermore, contrasting results have been reported regarding the roles of proteins in promoting the structural order of hydration water [20,21,22,23,24,25,27,28,29,33]. Specifically, some studies found that hydration water of amino acids or protein enhances the tetrahedral order of hydration water more than bulk water by using Raman multivariate curve resolution spectroscopy, differential scanning calorimetry, Fourier transform infrared spectroscopy, femtosecond surface sum frequency generation spectroscopy, and MD simulations [20,21,22,23,34], while other studies using neutron Brillouin measurements, wide-angle X-ray scattering, and MD simulations observed that protein reduces the tetrahedral order of hydration water [25,27,28,29,33]. Other parameters, such as d_5_ and local-structure index (LSI), account solely for the geometric arrangement of water molecules while neglecting one of the most critical factors in water structuring, namely, hydrogen bonding [22,24,30,31]. These limitations highlight the need for more robust and comprehensive approaches to investigate the impact of proteins on hydration water’s structure.

The dynamics of hydration water, including molecular vibrations and the restructuring of the hydrogen-bond network, as well as their coupling with protein fluctuations, have been the focus of numerous experimental and computational studies [10,35,36,37,38]. However, conflicting findings persist regarding the relationship between protein flexibility and hydration water dynamics [13,39,40,41,42]. Some studies have reported that higher protein flexibility is associated with reduced hydration water mobility [13,39,40], while others have observed the opposite, noting increased water mobility with greater protein flexibility [41,42]. These discrepancies highlight the complexity of protein–hydration water interactions, and the precise nature of their dynamic coupling remains unclear.

In this study, we performed all-atom molecular dynamics (MD) simulations of four well-characterized proteins to investigate the coupling between the protein structure and the structural order of hydration water. Our findings reveal that protein residues with greater flexibility or solvent exposure are associated with more disordered hydration water, resembling bulk water, whereas rigid and buried non-polar residues are surrounded by structurally ordered hydration shells. We further demonstrated that due to their strong hydrogen bonding and electrostatic interactions, charged residues exhibit the most disordered hydration water, while non-polar residues are surrounded by the most structurally ordered hydration water. By analyzing relaxation dynamics, we uncovered a positive correlation between the relaxation times of protein residues and their hydration water, indicating that slower (faster) protein relaxation is coupled with slower (faster) water relaxation. Furthermore, this coupling weakens with increasing residue flexibility or solvent exposure, with non-polar residues displaying the strongest coupling, and charged residues the weakest. To elucidate the mechanisms underlying these coupled fluctuations, we constructed scatter plots projected onto protein structural properties and hydration water order, revealing residue-specific coupling between protein and water structural ordering. These results provide new insights into the heterogeneous nature of protein–water interactions, advancing our understanding of how hydration water modulates protein stability, dynamics, and function.

## 2. Materials and Methods

### 2.1. Protein Selection and System Setup

We performed all-atom MD simulations on four well-characterized proteins: ubiquitin (PDB ID: 1UBQ [43]), lysozyme (PDB ID: 2VB1 [44]), alpha-chymotrypsin (PDB ID: 1YPH [45]), and ribonuclease A (PDB ID: 7RSA [46]). Each protein was placed in a cubic simulation box with a minimum distance of 2.5 nm from the protein to any box edge and solvated with TIP4P/2005 water molecules, following a previously described protocol [24]. To mimic physiological conditions, sodium and chloride ions were introduced into each protein system to neutralize the overall charge and achieve a salt concentration of 0.15 M. The proteins were described by the amber14sb force field [47], while ion parameters optimized for TIP4P/Ew were chosen due to their transferability to TIP4P/2005 [48]. For comparison, a pure water system (“Bulk”) was also constructed using the same force field parameters.

### 2.2. MD Simulations

All-atom MD simulations were performed using GROMACS 2023.2 [49]. After energy minimization by the steepest descent method, each system was pre-equilibrated in the NVT and NPT ensembles, with heavy protein atoms restrained by a harmonic potential, with the spring constant set to be 1000 kJ·mol^−1^·nm^−2^. Subsequently, two 500 ns production runs were conducted in the NPT ensemble without restraints. The temperature was maintained at 300 K using a v-rescale thermostat [50] with a coupling time constant of 0.2 ps, and the pressure was kept at 1.0 bar by the Parrinello–Rahman barostat [51], with a coupling time constant of 1.0 ps. Periodic boundary conditions were applied in all dimensions. Bonds involving hydrogen atoms were constrained with the LINCS algorithm [52], enabling a time step of 2 fs. Long-range electrostatic interactions were treated by the particle mesh Ewald method [53], and both electrostatic and van der Waals interactions used a 1.2 nm cutoff. The neighbor list, generated using a Verlet buffer tolerance of 0.005 kJ·mol^−1^·ps^−1^, was updated every 20 MD steps. During production, coordinates were saved every 10 ps, and statistical analyses were performed on the last 400 ns of each trajectory. After completing the 500 ns runs, an additional 5 ns production simulation (500–505 ns) was performed for each protein, with coordinates recorded every 0.1 ps to better capture the faster relaxation of the water and protein residues [2]. All the relaxation-time analyses were conducted on these 5 ns trajectories.

### 2.3. Calculating the Structural Order Parameter of Water

Orientational tetrahedral order q is one of the most widely used order parameter to characterize the local structural order of water, defined as follows [19,54]:

$$q=1-\frac{3}{8}\sum_{i=1}^{3}\sum_{j=i+1}^{4}{\left(cos\theta_{ij}+\frac{1}{3}\right)}^{2},$$

where θ_ij_ is the angle formed by two lines connecting the central water molecule and its nearest neighbors i and j, and the summation runs over all the combinations of the four nearest neighbors. It has a value of 0 and 1 for a random and a perfect tetrahedral configuration, respectively.

**Figure 1:**
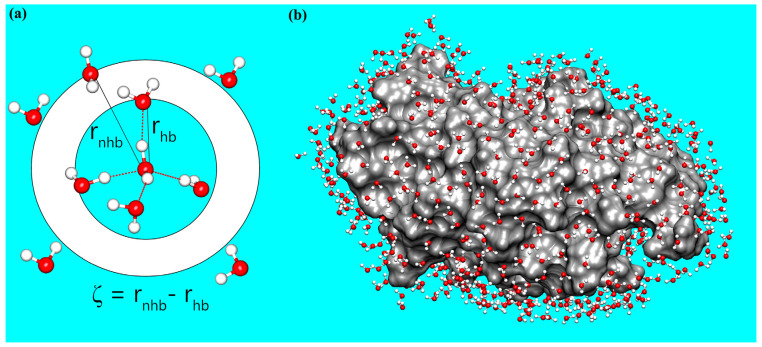
Schematic illustrations of the structural order parameter (ζ) and the protein hydration shell. (**a**) Definition of the structural order parameter ζ for a water molecule, calculated as the difference between the distance to the nearest non-hydrogen–bonded water (r_nhb_) and the distance to the furthest hydrogen–bonded water (r_hb_). (**b**) Visualization of the lysozyme protein (gray surface) with its hydration shell, defined as water molecules (CPK representation) within 5 Å of the protein carbon atoms. Here, oxygen and hydrogen atoms in a water molecule are shown in red and white, respectively.

Traditional structural order parameters, including tetrahedral order q, often fail to adequately capture the structural organization of protein hydration water [22,24]. Instead, we employed the structural order parameter ζ, proposed by Russo and Tanaka [18], which quantifies the local translational order of second-shell neighbors in water by measuring the extent to which non-hydrogen-bonded water molecules penetrate the first coordination shell, helping to distinguish between the two states (one with high translational symmetry, the other with low translational symmetry and a collapsed secondary hydration shell) that reflect water’s anomalies. Moreover, ζ can measure the depth of non-hydrogen-bonded water penetrating into the first hydrogen shell, thereby incorporating the hydrogen bond information, and has been successfully exploited to characterize the local structural ordering in pure water [18,55,56]. Specifically, ζ is defined for each water molecule as follows:

ζ = r_nhb_ − r_hb_,

where r_nhb_ is the distance (oxygen–oxygen) to the nearest non-hydrogen-bonded water, and r_hb_ is the distance to the furthest hydrogen-bonded water (Figure 1a). Two water molecules are considered hydrogen-bonded if the oxygen–oxygen distance is less than 3.5 Å and the oxygen–hydrogen–oxygen angle deviates by no more than 40° from linearity [24,57,58]. Small ζ values near zero indicate a weaker translational order of a water molecule, reflecting a disordered local environment in which a non-hydrogen-bonded water molecule penetrates into the first coordination shell. In contrast, larger ζ values denote enhanced translational order of a water molecule, consistent with a more complete hydrogen-bonding network.

### 2.4. Calculation of Root-Mean-Square Fluctuation (RMSF)

The RMSF value for each residue is a measure of the fluctuation (i.e., standard deviation) between the residue’s atomic positions and its time-averaged position in a trajectory of MD simulation. For atom i, the RMSF value was calculated using the expression given by

$$\sqrt{\frac{1}{T}\sum_{t=1}^{T}{\left(X_{i}(t)-\bar{X_{i}}\right)}^{2}}$$

where $X_{i}(t)$ represents the coordinates of atom i at time t, and $\bar{X_{i}}$ is its time average position over the simulation time of T. In this study, the RMSF was calculated using the program gmx rmsf from the GROMACS package [49]. In molecular dynamics simulations, a larger RMSF value for a residue indicates that it exhibits greater positional fluctuations around its average position, suggesting higher flexibility.

### 2.5. Calculation of Solvent-Accessible Surface Area (SASA)

The SASA is a measure of the surface area of a molecular structure that is accessible by the solvent molecules. Specifically, the SASA was calculated by considering the surface of the molecule as a series of points and then using a solvent probe sphere (a typical radius is 1.4 Å) to trace out the accessible surface area. In this study, the SASA was calculated using the program gmx sasa from the GROMACS package [49]. In molecular dynamics simulations, a larger SASA value for a residue indicates that it is more solvent-exposed.

### 2.6. Estimating the Relaxation Time

To examine the time-dependent correlation of ζ, the autocorrelation function C(τ) of ζ (Figure S5) was calculated using the expression given by

$$C\left(\tau \right)=\frac{<\left(\zeta \left(t\right)-\bar{\zeta }\right)\left(\zeta \left(t+\tau \right)-\bar{\zeta }\right)>}{<{\left(\zeta \left(t\right)-\bar{\zeta }\right)\;}^{2}>},$$

where $\bar{\zeta }$ is the mean value of $\zeta \left(t\right)$ over the simulation time (from t = 500 to 505 ns), τ is the lag time, and the angle brackets represent the ensemble or time average of the numerator and denominator. C(τ) captures how quickly the ζ value of a water molecule loses correlation with its initial state. For protein residues, the ζ value is not always continuously defined across the trajectory, leading to gaps in the calculated autocorrelation function C(τ). To address this issue, the autocorrelation function was divided into continuous time segments without interruptions. To quantify the decay rate of C(τ), the relaxation time τ_ζ_ for each continuous segment was estimated following a previous study by integrating C(τ) from τ = 0 to τ = T, where C(τ) first reaches 0.1, then is normalized by dividing by C(0) [59], with its expression given by

$$\tau_{\zeta }=\int_{0}^{T}\frac{C(\tau )}{C(0)}d(\tau ).$$

To ensure accuracy, segments in which the calculated relaxation time exceeded 10% of the corresponding segment length were considered unreliable and were discarded. Finally, the residue-specific relaxation time was computed using the mean value of all the valid segment relaxation times obtained after filtering.

We applied the same procedure to compute the autocorrelation function and relaxation time of the root mean square deviation for protein (RMSD), denoted τ_RMSD_. In both cases, larger (smaller) τ_ζ_ or τ_RMSD_ indicates a slower (faster) return to the initial hydrogen-bonding network for water or initial structure for the protein.

### 2.7. Calculating the Water Structural Ordering Velocity

To quantify how quickly ζ value changes, we calculated the derivative of ζ value with respect to a time interval (Δt) using:

$$v_{\zeta }=\frac{\zeta \left(t+\Delta t\right)-\zeta (t)}{\Delta t}$$

Because the relaxation time of ζ spans from 0.1 ps to a few picoseconds, we selected Δt = 0.1 ps and 1 ps to accurately capture the rate of ζ changing.

## 3. Results and Discussion

### 3.1. Residue-Specific Effects on Hydration Water Structure

The physical features (e.g., chain flexibility, surface size, exposure, and geometry) and chemical properties (e.g., polarity, charge, and hydrophobicity) of a protein surface are known to strongly influence the structural and dynamical characteristics of its hydration water [60,61,62,63]. These properties collectively shape the behavior of hydration water, ultimately impacting protein structural dynamics and function [60,63]. To explore how protein structure and dynamics couple with the structural order of hydration water, we performed all-atom MD simulations on four well-characterized proteins: ubiquitin, lysozyme, alpha–chymotrypsin, and ribonuclease A. We calculated the root-mean-square fluctuation (RMSF) and solvent-accessible surface area (SASA) for each protein residue, as well as the structural order parameter (ζ) for water molecules within the hydration shell (Figure S1) based on the two independent trajectories with time from t = 100 to 500 ns. Hydration water was defined geometrically as any water molecule with at least one atom within 5 Å of a protein carbon atom (Figure 1b) [1]. For each residue, we identified its “contact water” as the subset of hydration water molecules closest to that residue. The ζ value for each residue was then calculated by averaging ζ over all its contact water molecules. This approach ensured that only surface residues, which directly interact with hydration water, were assigned ζ values.

**Figure 2:**
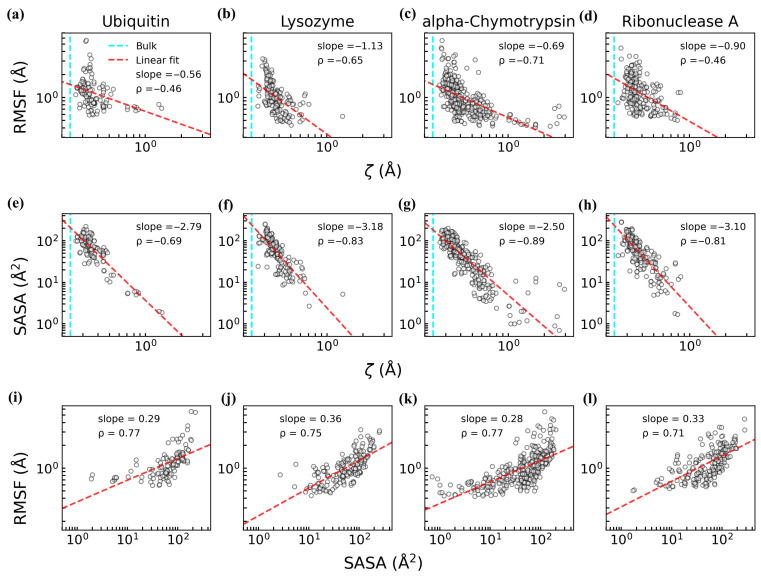
Correlations among protein structural properties, dynamical properties, and the structural order parameter (ζ) of hydration water for four proteins (ubiquitin, lysozyme, alpha–chymotrypsin, and ribonuclease A) based on the two independent trajectories with time from t = 100 to 500 ns. The correlation is quantified by Spearman correlation coefficient (*ρ*) and a linear fitting (red dashed line), with the slope shown on each subplot. (**a**–**d**) Correlation between RMSF and the ζ value of protein residues. (**e**–**h**) Correlation between SASA and the ζ value of protein residues. (**i**–**l**) Correlation between RMSF and SASA value of protein residues. In panels (**a**–**h**), the cyan dashed lines denote the mean ζ value of bulk water (0.24 Å). The ζ value assigned to each residue is the average of the ζ values for the water molecules in direct contact with that residue.

Our analysis revealed negative correlations between RMSF and ζ across all four proteins (Figure 2a–d), according to the results of Spearman correlation coefficients (*ρ*) and linear fitting (red dashed line), indicating that residues with higher flexibility are surrounded by more disordered hydration water, while rigid residues are associated with more ordered hydration shells. Notably, the hydration water near these residues exhibited greater structural order compared to bulk water (cyan dashed lines in Figure 2a–d), consistent with previous findings that proteins promote local water structuring [24]. Similarly, a negative correlation was observed between SASA and ζ (Figure 2e–h), highlighting the tight coupling between protein structural features and hydration water order. These relationships can be attributed to the strong correlation between SASA and RMSF (Figure 2i–l): residues with higher flexibility typically have larger solvent-exposed areas [64,65], which disrupt the structural order of nearby water.

**Figure 3:**
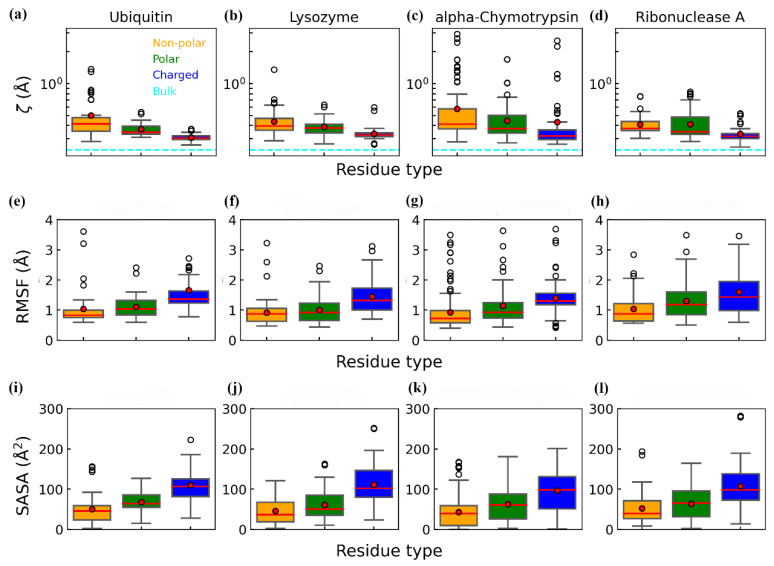
Distributions of ζ, RMSF, and SASA values for non-polar, polar, and charged residues across the four proteins based on the two independent trajectories with time from t = 100 to 500 ns. (**a**–**d**) Distributions of ζ values. (**e**–**h**) Distributions of RMSF values. (**i**–**l**) Distributions of SASA values. Glycine residues were excluded from the analysis. Red dots and red lines indicate the mean and median values of each distribution, respectively. In panels (**a**–**h**), the cyan dashed lines denote the mean ζ value of bulk water (0.24 Å).

To examine how residue chemical properties influence the structural order of hydration water, we analyzed the distributions of ζ, RMSF, and SASA across three residue categories (non-polar, polar, and charged, excluding glycine) (Figure 3). The mean (red point) and median (red line) values of each distribution reveal that charged residues generally exhibit the largest RMSF and SASA, alongside the smallest ζ. In contrast, non-polar residues show the smallest RMSF and SASA but the highest ζ, whereas polar residues occupy intermediate ranges for these properties. These trends indicate that more rigid, less solvent-exposed, non-polar residues correlate with more structurally ordered hydration water, consistent with previous findings that hydrophobic surfaces promote interfacial water ordering [66,67,68]. In contrast, charged residues, being more flexible and exposed, are associated with more disordered hydration water closely resembling the bulk. Water molecules generally form three to four hydrogen bonds in liquid water [17], and near charged residues, they can still form hydrogen bonds with neighboring water molecules while also interacting with the charged side chains in a manner akin to the bulk phase [69]. A previous computational study also showed that the more frequent interaction between water molecules and the oxygen atoms, probably from charged residues, decreases the deviation of ordering from bulk water [42]. This mechanism underlies a tendency that a more disordered hydration shell is more likely to be observed around charged residues, aligning with experimental reports that charged amino acids enhance protein solubility [70,71,72]. Although the average ζ values differ across residue types, the substantial overlap in their distributions reflects significant intra-type variability in hydration water’s structure. This variability arises from differences in local residue flexibility and solvent exposure, as quantified by RMSF and SASA. Thus, the observed hydration water ordering is not solely determined by residue chemical identity but is strongly influenced by physical context, resulting in a complex and heterogeneous hydration shell with residue-specific characteristics.

**Figure 4:**
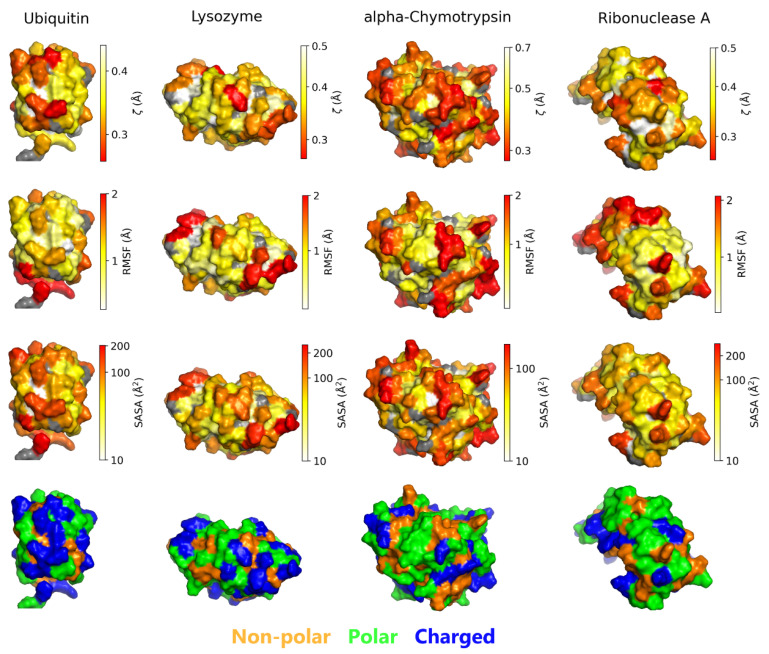
Visualization of ζ, RMSF, SASA, and residue type distributions on the surfaces of the four proteins based on their two independent trajectories with time from t = 100 to 500 ns. Each column represents the protein PDB structure, with residues colored according to ζ (**top row**), RMSF (**second row**), SASA (**third row**), and residue type (**bottom row**). Residues colored in gray indicate that ζ could not be calculated.

To visualize how physical and chemical heterogeneity of protein influences hydration water’s structure, we mapped ζ, RMSF, SASA, and residue types on the surfaces of the four proteins (Figure 4; a similar representation using a histogram is shown in Figure S4). Residues with similar coloring in the upper three rows occupy analogous regions in each protein, reflecting the positive correlation between RMSF and SASA, as well as their negative correlation with ζ. In addition, the spatial distributions of these three properties closely align with the arrangement of residue types on the protein surface.

Collectively, our results show that protein surface heterogeneity governs variations in the structural ordering of the hydration shell: rigid, buried non-polar residues tend to promote a more ordered water network, whereas flexible, exposed charged residues yield water closer to the bulk state. Unlike non-polar residues, which facilitate ordered hydration primarily by minimizing interfacial entropy, charged residues perturb water through hydrogen bonding and electrostatic interactions, ultimately producing a more dynamically disordered, bulk-like hydrogen-bond network.

### 3.2. Dynamics of Protein–Water Relaxation

**Figure 5:**
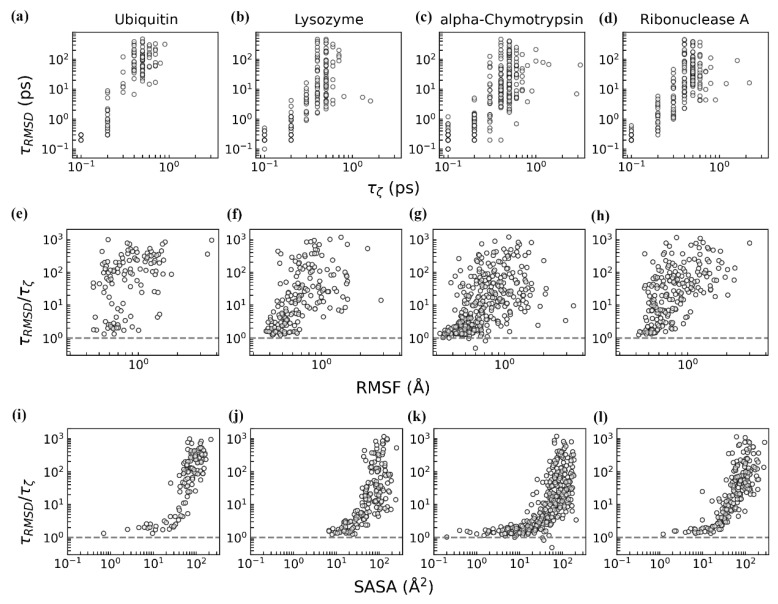
Correlations between the relaxation times of protein residues and their hydration water for four proteins. (**a**–**d**) Correlation between the RMSD relaxation time of individual residues (τ_RMSD_) and the relaxation time of the structural order parameter ζ in their hydration water molecules (τ_ζ_). (**e**–**h**) Influence of residue flexibility (RMSF) on the coupling ratio, τ_RMSD_/τ_ζ_. (**i**–**l**) Influence of residue solvent exposure (SASA) on the coupling ratio, τ_RMSD_/τ_ζ_. In panels (**e**–**l**), the gray dashed lines mark the point at which τ_RMSD_/τ_ζ_ = 1. All the relaxation times were estimated based on their two independent trajectories, with time from t = 500 to 505 ns.

To characterize the coupled dynamics of individual protein residues and their hydration water, we calculated the relaxation times of residue RMSD (τ_RMSD_) and the water structural order parameter ζ (τ_ζ_) (see Materials and Methods for details). Across all four proteins, hydration water exhibited relaxation times (τ_ζ_) ranging from 0.1 to 3 ps, while protein residues showed significantly longer relaxation times (τ_RMSD_) of 0.1–500 ps (Figure 5a–d). These timescales align with established experimental and computational findings [2,6,63,73,74], where water (e.g., through rotational, re-orientational, translational relaxation, and hydrogen-bond dynamics) relaxes more rapidly than the slower structural fluctuations of the protein. We observed a positive correlation between τ_ζ_ and τ_RMSD_ across all the residues (Figure 5a–d), indicating that slower-relaxing residues are coupled with slower-relaxing hydration water. Thus, residues exhibiting slower (faster) relaxation are associated with slower (faster) relaxation of the structural order of their hydration water, consistent with earlier findings of protein–water dynamical interplay [6,63,74]. Notably, prolonged relaxation times do not reflect reduced mobility but rather extended recovery periods following perturbations. Consistent with this interpretation, residues exhibiting greater flexibility or solvent exposure demonstrated extended relaxation times for both structural fluctuations and adjacent water dynamics (Figure S2).

To quantify the degree of protein–water coupling, we analyzed the ratio of relaxation times, τ_RMSD_/τ_ζ_ (Figure 5e–l) [74]. Larger values of this ratio indicate greater disparity between protein and water relaxation timescales, reflecting weaker coupling. When τ_RMSD_/τ_ζ_ ≈ 1, the protein and water relax synchronously. Across all the residues, the ratio predominantly exceeds 1 (gray dashed line in Figure 5e–l), confirming that protein relaxation is generally slower than that of hydration water [2,74]. This observation supports the “slaving” water model, wherein the slower protein residue motions are driven by the faster fluctuations of hydration water [74,75]. We further investigated how residue flexibility (RMSF) and solvent exposure (SASA) influence the coupling degree (Figure 5e–l). The results indicate that higher RMSF or SASA correlates with smaller coupling (i.e., larger τ_RMSD_/τ_ζ_), implying that flexible, more solvent-exposed residues respond less synchronously with nearby water. Their increased solvent exposure broadens the interaction network, which may reduce the extent to which water dynamics drive residue relaxation.

**Figure 6:**
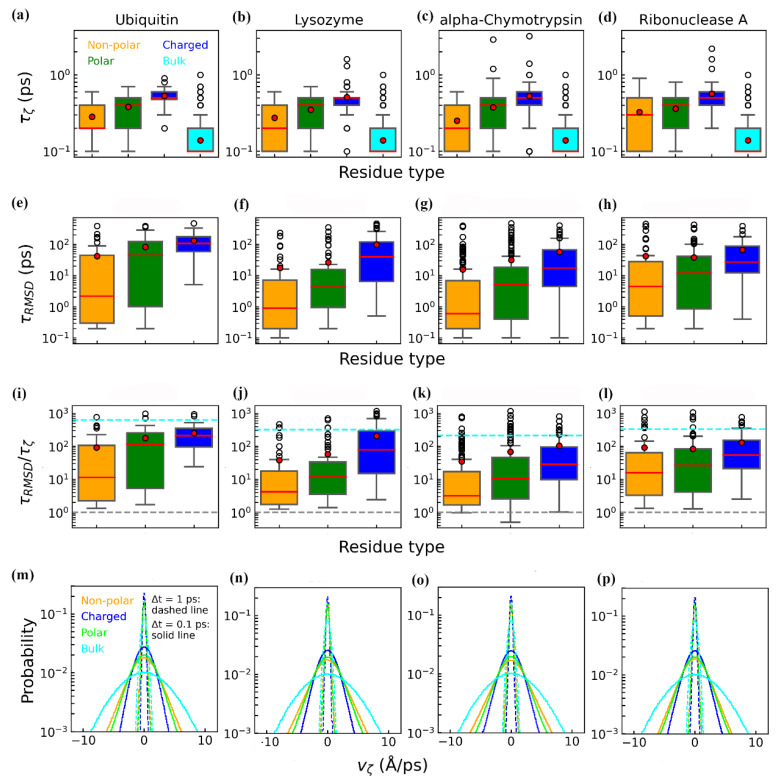
Distributions of relaxation times and coupling ratios for non-polar, polar, and charged residues across four proteins. (**a**–**d**) Distributions of the ζ relaxation time (τ_ζ_). (**e**–**h**) Distributions of the RMSD relaxation time (τ_RMSD_). (**i**–**l**) Distributions of the coupling ratio τ_RMSD_/τ_ζ_. In panels (**i**–**l**), the gray dashed lines indicate τ_RMSD_/τ_ζ_ = 1, and the cyan dashed lines represent the ratio of the mean relaxation times for all the residue types relative to bulk water. (**m**–**p**) Probability distributions of the velocity of ζ (v_ζ_) for non-polar, polar, and charged residues. The color schemes for residue types are the same as those in Figure 3 and Figure 4. In each box plot, the red point denotes the mean value, and the red line indicates the median value. All the relaxation times were estimated based on their two independent trajectories, with time from t = 500 to 505 ns.

To explore how residue chemical properties modulate hydration-water dynamics, we examined the distributions of relaxation times for both ζ (Figure 6a–d) and RMSD (Figure 6e–h) across non-polar, polar, and charged residues. Overall, each protein exhibits similar trends for these three residue types, and all residue types exhibited relaxation times longer than those of bulk water, consistent with experimental and computational evidence of reduced water mobility near protein surfaces [9,74,76]. This slowdown arises, in part, from fewer and less flexible hydrogen-bonding configurations compared to bulk water [9]. Among the residue types, charged residues displayed the longest relaxation times for both ζ (~0.5 ps) and RMSD (~100 ps), while non-polar residues exhibited the shortest (~0.3 ps for ζ and ~30 ps for RMSD). Polar residues fall between these extremes (~0.4 ps for ζ and ~45 ps for RMSD). These trends suggest that strong electrostatic interactions at charged sites prolong water and residue relaxation, whereas weaker interactions at non-polar sites facilitate faster structural reorganization. Non-polar residues and their surrounding hydration water exhibit faster relaxation than other residue types, likely due to the weaker interactions experienced by water near buried and rigid non-polar surfaces [72]. In contrast, charged residues form stable hydrogen-bonding and electrostatic networks, slowing both residue and water dynamics [74]. Additionally, slower water dynamics near charged groups are attributed to extended hydrogen-bond lifetimes, which reduce fluctuations in local water density [40,77].

To further assess residue-specific coupling, we calculated the ratio τ_RMSD_/τ_ζ_ for each residue and compared these values across the three residue classes (Figure 6i–l). Non-polar residues exhibited the smallest mean ratio (~100), indicating the strongest coupling, followed by polar residues (~110). Charged residues showed the largest ratio (~200), approaching the coupling observed between residues and bulk water beyond the hydration shell (cyan dashed lines in Figure 6i–l). This reduced coupling for charged residues likely arises from the pronounced disparity between their relaxation times (τ_RMSD_) and those of hydration water (τ_ζ_). Charged residues form strong hydrogen bonds and electrostatic interactions with both water and neighboring residues, prolonging structural relaxation. Additionally, their higher solvent exposure and structural flexibility further hinder rapid relaxation (Figure S2). Consequently, charged residues display longer relaxation times overall. While water near charged residues also experiences slower dynamics, the effect is less pronounced than for the residues themselves. This differential slowdown results in stronger decoupling of residue and water relaxation times for charged residues compared to non-polar and polar types.

To further quantify how rapidly the structural order of hydration water responds to protein fluctuations, we calculated the rate of change of ζ, denoted as v_ζ_, for each residue (Figure 6m–p). We evaluated v_ζ_ using two time intervals: 0.1 ps (solid lines) and 1 ps (dashed lines). Since 1 ps exceeds the typical ζ relaxation time for most residues (Figure 6a–d), the resulting distributions are narrower and similar in width across all the residue types. Therefore, our analysis primarily focuses on the 0.1 ps distributions. Across the four proteins, consistent trends emerged: charged residues (blue lines) exhibit the narrowest and highest probability peaks, followed by polar residues with moderately broad peaks, while non-polar residues present the broadest and lowest peaks. To quantify the rate of ζ changes, we used the halfwidth of each v_ζ_ distribution. The mean halfwidth values for the non-polar, polar, and charged residues are 2.3, 2.1, and 1.7 Å/ps, respectively. These findings align with the shorter relaxation times observed for non-polar residues (Figure 6a–d), indicating that their hydration water undergoes faster structural rearrangements compared to polar or charged residues.

Collectively, our results demonstrate that slower (faster) residue relaxation is coupled with slower (faster) hydration-water relaxation. However, the degree of coupling between protein residues and hydration water is modulated by both the physical and chemical properties of the protein surface. Charged residues, which are more solvent-exposed, form stronger hydrogen-bonding and electrostatic interactions with surrounding water, thereby delaying water relaxation and resulting in the weakest coupling. In contrast, non-polar residues, which are generally more rigid and buried, exhibit weaker interactions with water and display tighter coupling, accompanied by faster overall dynamics.

### 3.3. Coupled Protein–Water Fluctuations

**Figure 7:**
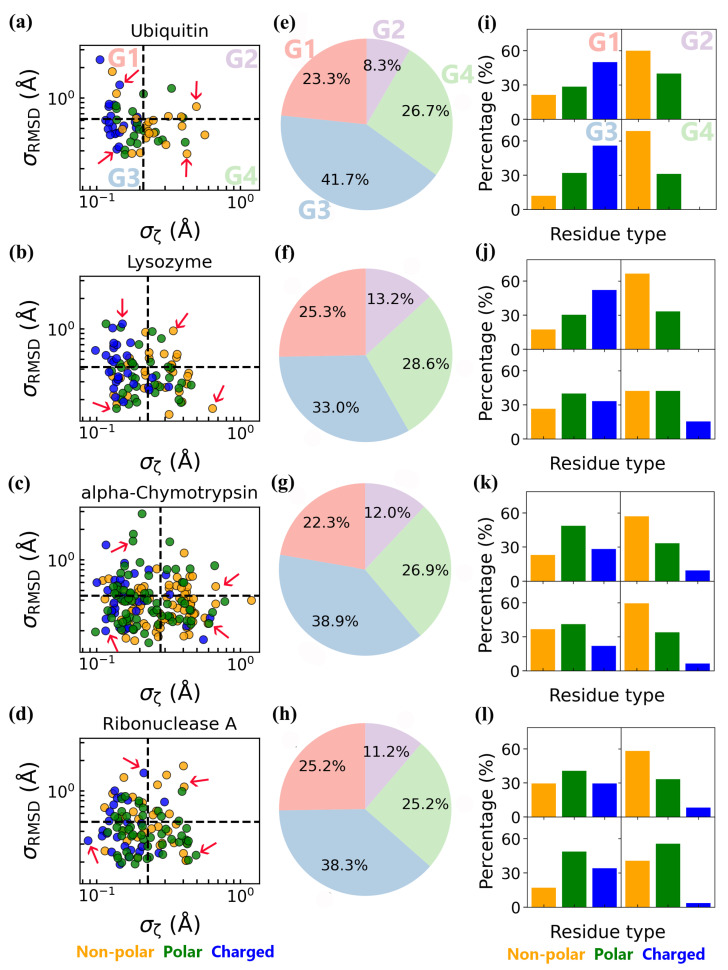
Classification of protein residues by their standard deviations in ζ (σ_ζ_) and RMSD (σ_RMSD_) across four proteins based on their two independent trajectories, with time from t = 500 to 505 ns. (**a**–**d**) Residues are divided into four groups (G1, G2, G3, G4) based on the mean values of σ_ζ_ and σ_RMSD_ (black dashed lines). The red arrows denote the representative residues in scatter plots (Figure S3) showing residue-specific coupling of fluctuation between protein and water. (**e**–**h**) Proportions of residues in each of the four groups. (**i**–**l**) Fractions of non-polar, polar, and charged residues within the four groups.

Our abovementioned findings indicate that protein residues and their hydration water exhibit coupled thermodynamics and dynamics, with the coupling degree influenced by the flexibility, solvent exposure, and chemical properties of surface residues. As a result, the degree of coupling varies across different regions of the protein surface. To elucidate the heterogeneity of coupled protein–water dynamics, we analyzed the standard deviations of residue RMSD (σ_RMSD_) and hydration water structural order ζ (σ_ζ_) across all the surface residues (Figure 7a–d). The residues were classified into four distinct groups (G1, G2, G3, and G4) based on whether σ_RMSD_ and σ_ζ_ exceeded their respective mean values (black dashed lines in Figure 7a–d) based on their two independent trajectories, with time from t = 500 to 505 ns. Specifically, residues in G1 and G4 exhibited elevated σ_RMSD_ (high structural flexibility) and σ_ζ_ (high water order fluctuations) relative to G3, respectively, while those in G2 showed elevated standard deviations for both parameters. Notably, G3, which contains the largest fraction of residues, displayed relatively stable structures and hydration water order (Figure 7e–h), underscoring their role in maintaining global protein stability.

We also analyzed the fraction of non-polar, polar, and charged residues in each of the four groups (G1–G4) and compared these fractions to the overall distribution of the residue types on the protein surface (Table S1). Although most surface residues in each protein are either charged or polar, the breakdown by group reveals striking differences (Figure 7i–l, Table S2). Non-polar residues predominated in G2 and G4, where σ_ζ_ is relatively high, consistent with the wider distribution of ζ values for non-polar residues in Figure 3a–d. In contrast, charged and polar residues were more abundant in G1 and G3, aligning with their relatively larger RMSF values (Figure 3e–h).

To resolve the mechanisms of coupled fluctuations between protein residues and their hydration water, we constructed scatter plots as functions of RMSD and ζ for each group (Figure S3). These scatter plots, based on trajectories from t = 500 to 505 ns, effectively capture the rapid motions of protein residues and their surrounding hydration water molecules. As a result, they are suitable for illustrating coupled fluctuations between the protein and water on short timescales, and they provide meaningful insight into the residue-specific variations in the degree of protein–water coupling, especially in regimes where the relaxation times of protein and hydration water are comparable. This analysis complements our findings based on the relaxation time ratio τ_RMSD_/τ_ζ_, highlighting residues where fast, synchronous dynamics emerge from strong coupling with hydration water.

Notably, the scatter plots for residues within the same group exhibit characteristic profiles. For instance, the scatter plots of G1 and G4 residues are narrow and elongated along the RMSD and ζ-value axes, respectively, whereas the G2 and G3 residues display relatively isotropic, yet size-differentiated basins. Specifically, G1 residues have high structural flexibility paired with relatively rigid hydration water order, while G4 residues maintain more rigid structures but higher structural order flexibility in their hydration water. In contrast, G3 residues show stability in both structure and water ordering, whereas G2 residues exhibit flexibility in both.

Collectively, our scatter plot analysis reveals that protein surfaces are mosaics of dynamically distinct regions. Residue-specific coupling mechanisms, dictated by flexibility, solvent exposure, and chemical properties, govern whether water or protein motions act as the primary driver of structural transitions. This hierarchical dynamic interplay underscores the critical role of hydration water as both a mediator and modulator of protein conformational landscapes.

## 4. Conclusions

In this study, we investigated how protein structure influences the thermodynamics and dynamics of its hydration water using a recently developed structural order parameter (ζ) [18]. From a thermodynamic standpoint, our findings reveal that proteins generally enhance the local structural order of hydration water, consistent with previous studies [24]. However, this effect is highly heterogeneous across the protein surface: residues with greater flexibility or solvent exposure are associated with more disordered hydration water, resembling the bulk phase, while rigid and buried non-polar residues promote more ordered hydration shells. These differences arise from the distinct interactions between water and different residue types. Charged residues, with their strong hydrogen-bonding and electrostatic interactions, disrupt the local water network, leading to a more disordered, bulk-like hydration shell. In contrast, non-polar residues facilitate stronger water–water hydrogen bonding, reinforcing structural order [66,67,68,69].

To make a comparison of the ζ with q (one of the most widely used order parameters) of water to further understand the structural order of hydration water, we calculated the tetrahedrality parameter q (see Material and Methods section) for hydration water around all the surface residues in each of the four proteins. We then generated scatter plots of the per-residue ζ versus q (Figure S6). The calculated mean q value (the cyan dashed lines in the horizontal direction in Figure S6) for bulk water in our simulations is about 0.66, in agreement with previous studies [78,79]. This confirms that our simulation correctly reproduced the known tetrahedral characteristic of bulk water. In addition, the calculated tetrahedrality q values for hydration water around the surface residues of the four proteins were consistently lower than the corresponding bulk water value, which aligns well with some previous computational and experimental studies [26,27,32,79]. However, some other studies observed contrasting results [20,21,22,23,34]. In addition, to investigate the ζ–q correlation, we performed linear fitting (red dashed line in Figure S6) and calculated Spearman correlation coefficients (*ρ*) between ζ and q for the residues of each protein. In all four proteins, we observed a consistent modest negative correlation between ζ and q, which indicates that ζ and q capture distinct aspects of the hydration-water structure. Notably, here are a few surface residues with q < 0. The order parameter q is nominally defined from 0 (ideal gas) to 1 (perfect tetrahedron) [78]. q < 0 might arise from exceptionally constrained geometries and orientations of hydration water, which have been reported in octanol–water and methanol–water interfacial systems [80,81]. q is an orientational order parameter, and the rotational symmetry of interfacial water will inevitably be disrupted in the presence of protein. It makes many interfacial water molecules have less than four neighbors in the first coordination shell, leading to inaccurate calculation of the orientational ordering q of interfacial water, and even causing negative q with a confined angle of neighboring water. These negative-q cases fall outside the usual 0 to 1 range and suggest that tetrahedral order parameter q may not fully characterize the structural order of hydration water. ζ quantifies the local translational order of second-shell neighbors in water by measuring the extent to which non-hydrogen-bonded water molecules penetrate the first coordination shell. Hence, ζ is not much influenced by the disruption of rotational symmetry of neighbors in the first shell like q and is more suitable to quantify residue-specific variations of the structural order of hydration water.

From a dynamic perspective, we observed that charged residues exhibit the longest relaxation times for both ζ and RMSD, while non-polar residues show the shortest relaxation times. Polar residues fall between these extremes, displaying moderate relaxation times. Importantly, we found a positive correlation between the relaxation times of protein residues and their hydration water, indicating that slower (or faster) relaxation of water is coupled with slower (or faster) relaxation of the corresponding residues. To quantify the degree of coupling between the protein residues and hydration water, we calculated the relaxation time ratio (τ_RMSD_/τ_ζ_). Most residues exhibited a ratio greater than 1, indicating that protein residues generally relax more slowly than their hydration water. This finding supports the “slaving model”, which posits that protein residue motions are influenced and controlled by hydration water fluctuations [74,75].

We found that the degree of coupling between the protein residues and hydration water varies significantly depending on the flexibility, solvent exposure, and chemical properties of the residues. Charged residues, with their higher solvent exposure and stronger interactions with water, exhibit the weakest coupling, while non-polar residues, which are generally more rigid and buried, show the strongest coupling. This reduced coupling for charged residues can be attributed to their greater solvent exposure, which allows them to form stronger hydrogen-bonding and electrostatic interactions with neighboring hydration water. These interactions hinder the relaxation of water molecules. Additionally, the higher flexibility and exposure of charged residues enable them to form stronger interactions with adjacent oppositely charged residues and/or hydration water, further delaying their relaxation back to the equilibrium. This heterogeneity in coupling strength underscores the complex and diverse nature of protein–water interactions across the protein surface.

To further explore these coupled fluctuations, we generated scatter plots as functions of RMSD and ζ. These scatter plots highlight the dynamic interplay between protein residues and their hydration water, demonstrating that coupled fluctuations can proceed via different mechanisms depending on local residue–water interactions. Overall, our results emphasize the importance of residue-specific analysis in understanding protein–water coupling. Unlike previous studies that focused primarily on global hydration shell behavior or average water dynamics, our approach resolves localized dynamic interactions that govern protein conformational plasticity. The integration of local structural order parameters, relaxation time ratios, and short-timescale scatter plots enables us to dissect hierarchical coupling mechanisms at the molecular level. In doing so, we uncover how hydration water not only reflects but also actively modulates the conformational dynamics of proteins, thereby acting as both a mediator and modulator of protein function.

Our findings also reveal that proteins exert distinct effects on the thermodynamics and dynamics of bulk water. Charged residues induce the least perturbation to the thermodynamics of bulk water, as the structural order of hydration water near these residues closely resembles that of bulk water. However, they significantly perturb water dynamics, as evidenced by the prolonged relaxation times of hydration water near charged residues. This apparent contradiction between thermodynamic and dynamic perspectives underscores the complex nature of hydration water and its role in protein behavior [61,82]. While this study focuses on the emergent coupling between hydration water and protein residues in their native conformational and electrostatic states, a more direct assessment of the role of electrostatics could be achieved by selectively neutralizing charged residues in simulations. Such an approach may help disentangle the specific contribution of electrostatic interactions to hydration-shell ordering and relaxation dynamics. However, we note that neutralizing charged residues can also alter key intra-protein interactions (e.g., salt bridges) and affect local structure and flexibility, making it difficult to isolate electrostatics from other confounding effects. Additionally, the influence of charge neutralization would need to be tested across multiple systems and residue environments to yield statistically robust conclusions. Despite these challenges, we recognize the value of this direction and plan to pursue such perturbative strategies in future work to more precisely quantify the electrostatic contributions to protein–water coupling.

In summary, our study provides new insights into the coupling between protein residues and hydration water, both thermodynamically and dynamically. By revealing how residue flexibility, solvent exposure, and chemical properties influence the structural order and relaxation dynamics of hydration water, our findings shed light on the heterogeneous nature of protein–water interactions. These results advance our understanding of the critical role that hydration water plays in modulating protein behavior, offering a foundation for future research in protein folding, stability, and function.

## Data Availability

The necessary files for setting up the GROMACS (version 2023.2) simulations and analysis pro-grams/scripts are publicly available at https://osf.io/skb5h/ (accessed on 1 April 2025). The data that support the findings of this study are available within the article and its Supplementary Material and from the corresponding author upon reasonable request.

## Supplementary Materials

The following supporting information can be downloaded at: https://www.mdpi.com/article/10.3390/biom15050660/s1, Table S1. Composition of surface residues by type (non-polar, polar, and charged) for the four proteins; Table S2. Distribution of residue types (non-polar, polar, and charged) across the four groups (G1, G2, G3 and G4) for the four proteins. Groups are defined based on the standard deviations of RMSD and ζ values (see Figure 7 in the main text). The group with the highest proportion for each residue type is highlighted in red. The results indicate that non-polar residues predominantly distribute in G4, whereas polar and charged residues are more likely to be found in G3; Figure S1. Time evolution of ζ, RMSD and SASA values during the simulation period from 100 to 505 ns for the four proteins; Figure S2. Correlation between the structural and dynamical properties of protein residues (RMSF and SASA) and the relaxation times of residue fluctuations (τ_RMSD_) or hydration water structural order (τ_ζ_) for the four proteins; Figure S3. The scatter plots for some representative residues from each group (G1, G2, G3, G4) across the four proteins based on their two independent trajectories with time from t = 500 to 505 ns, showing residue-specific coupling of fluctuation between protein and structural order of hydration water; Figure S4. Histogram of the ζ values for all surface residues across the four proteins obtained from two independent simulation trajectories over the time interval t = 100 to 500 ns. For the spatial distribution of ζ values mapped onto the protein structures, refer to Figure 4 in the main text; Figure S5. Scatter plots of the autocorrelation functions C(τ) for RMSD and ζ, along with their corresponding relaxation times (τ_ζ_ or τ_RMSD_), for a subset of residues from the four proteins; Figure S6. Correlations between the two structural order parameters (ζ and q) of hydration water for each surface residues from the four proteins (ubiquitin, lysozyme, α-chymotrypsin, and ribonuclease A) The correlation is quantified by spearman correlation coefficient (*ρ*) and a linear fitting (red dashed line) with slope and ρ shown on each subplot. Two blue dashed lines in the vertical and horizontal directions on each subplot denote the mean ζ (0.24 Å) and mean q value (0.66) of bulk water, respectively.
