# Supplementary material for: Residue-Specific Structural and Dynamical Coupling of Protein and Hydration Water Revealed by Molecular Dynamics Simulations
Source: Biomolecules. 2025 May 2;15(5):660. doi: 10.3390/biom15050660 (PMC12108842; doi:10.3390/biom15050660)
Supplement: Supplementary file 1 [file biomolecules-15-00660-s001.zip › biomolecules-3563173-supplementary.pdf]

Supplemental Online Material for

Residue-Specific Structural and Dynamical Coupling of Protein and Hydration Water Revealed by Molecular Dynamics Simulations

Shuai Wang [1,2], Jun Gao [1] and Xiakun Chu [2,3,4,*]

[1] College of Informatics, Huazhong Agricultural University, Wuhan 430070, China;
shuaiwang@hkust-gz.edu.cn (S.W.); gaojun@mail.hzau.edu.cn (J.G.)
[2] Advanced Materials Thrust, Function Hub, The Hong Kong University of Science and Technology
(Guangzhou), Guangzhou 511400, China
[3] Guangzhou Municipal Key Laboratory of Materials Informatics, The Hong Kong University of Science
and Technology (Guangzhou), Guangzhou 511400, China
[4] Division of Life Science, The Hong Kong University of Science and Technology, Clear Water Bay,
Hong Kong SAR 999077, China
* Correspondence: xiakunchu@hkust-gz.edu.cn

Additional tables

Table S1. Composition of surface residues by type (non-polar, polar, and charged) for the four proteins.

Protein	Non-polar (%)	Polar (%)	Charged (%)
Ubiquitin	33.3	31.7	35.0
Lysozyme	34.1	37.4	28.6
alpha-Chymotrypsin	42.3	40.0	17.7
Ribonuclease A	30.8	46.7	22.4

Table S2. Distribution of residue types (non-polar, polar, and charged) across the four groups (G1, G2, G3 and G4) for the four proteins. Groups are defined based on the standard deviations of RMSD and ζ values (see **Figure 7** in the main text). The group with the highest proportion for each residue type is highlighted in red. The results indicate that non-polar residues predominantly distribute in G4, whereas polar and charged residues are more likely to be found in G3.

Protein	Residue type	G1 (%)	G2 (%)	G3 (%)	G4 (%)
Ubiquitin	Non-polar	15.0	15.0	15.0	**55.0**
	Polar	21.1	10.5	**42.1**	26.3
	Charged	33.3	0.0	**66.7**	0.0
Lysozyme	Non-polar	12.9	25.8	25.8	**35.5**
	Polar	20.6	11.8	**35.3**	32.3
	Charged	**46.1**	0.0	38.5	15.4
alpha-Chymotrypsin	Non-polar	12.2	16.2	33.8	**37.8**
	Polar	27.1	10.0	**40.0**	22.9
	Charged	35.5	6.4	**48.4**	9.7
Ribonuclease A	Non-polar	24.3	21.2	21.2	**33.3**
	Polar	22.0	8.0	**40.0**	30.0
	Charged	33.3	4.2	**58.3**	4.2

Additional figures

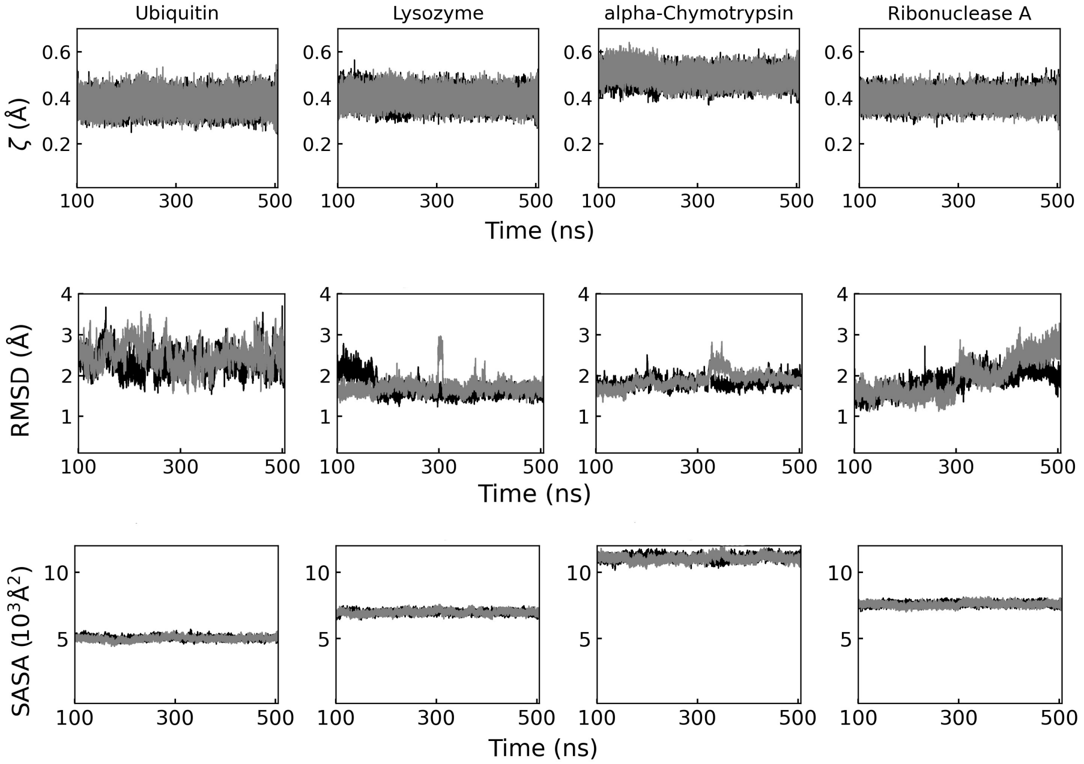

Figure S1. Time evolution of ζ, RMSD and SASA values during the simulation period from 100 to 505 ns for the four proteins.

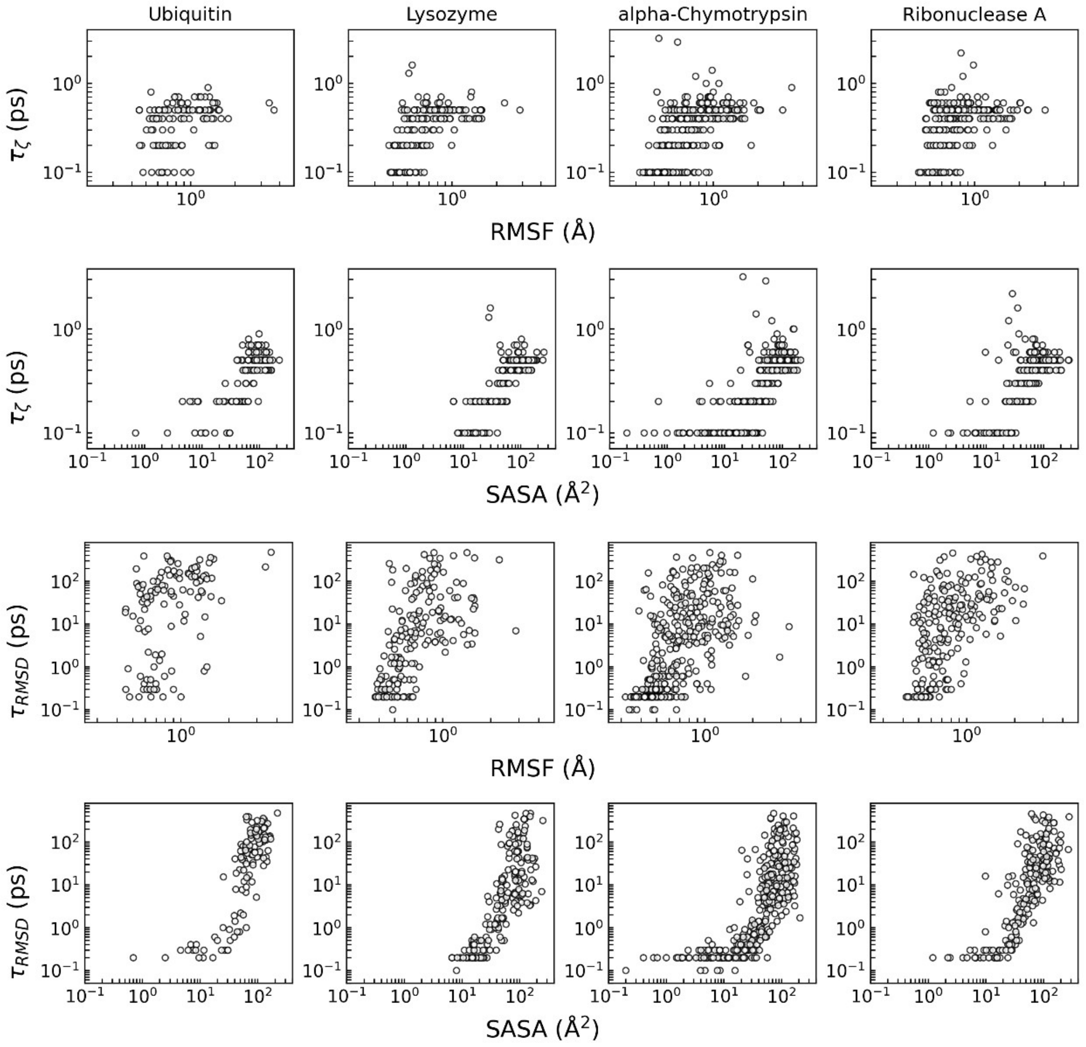

Figure S2. Correlation between the structural and dynamical properties of protein residues (RMSF and SASA) and the relaxation times of residue fluctuations (τ_{RMSD}) or hydration water structural order (τ_ζ) for the four proteins.

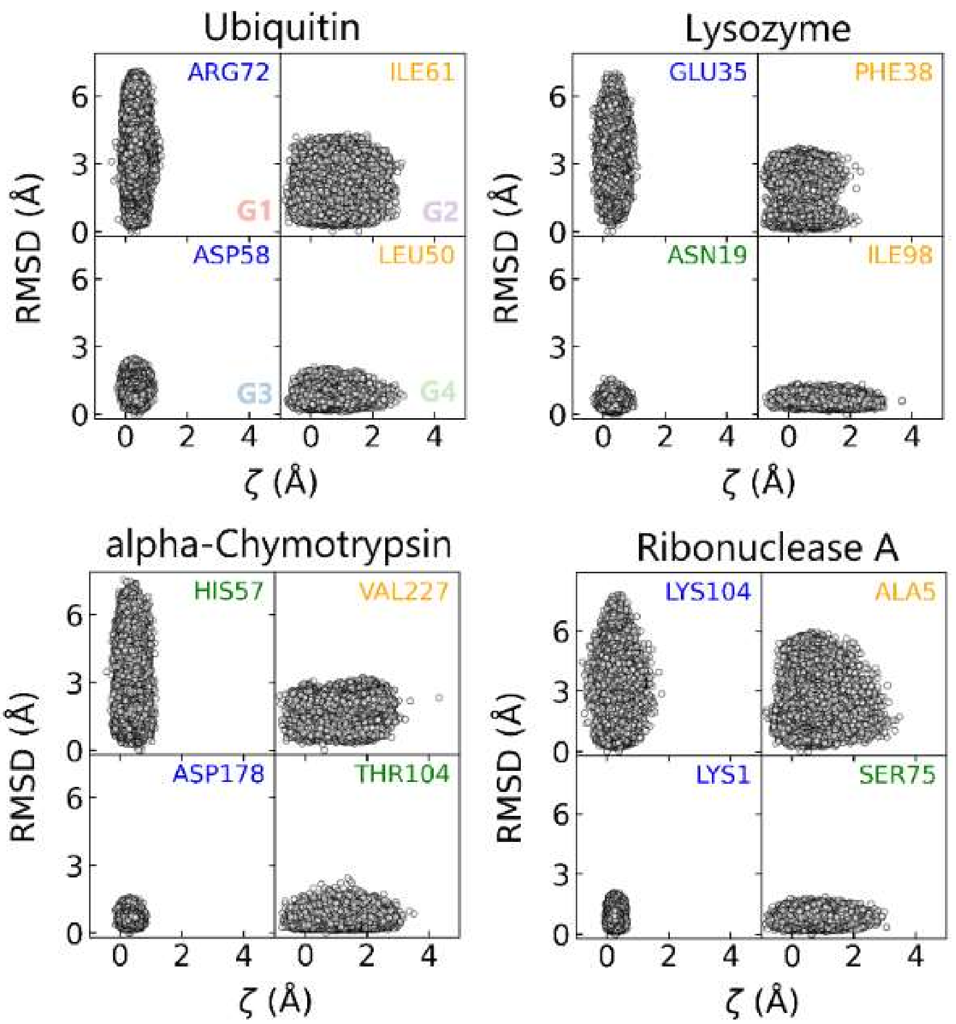

Figure S3. The scatter plots for some representative residues from each group (G1, G2, G3, G4) across the four proteins based on their two independent trajectories with time from t=500 to 505 ns, showing residue-specific coupling of fluctuation between protein and structural order of hydration water.

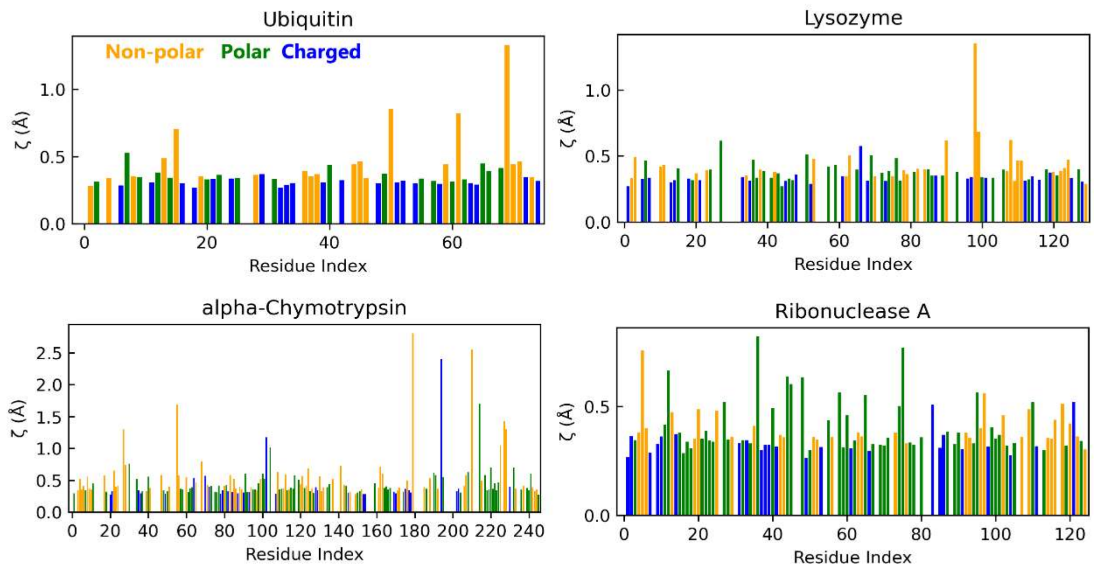

Figure S4. Histogram of the ζ values for all surface residues across the four proteins obtained from two independent simulation trajectories over the time interval t = 100 to 500 ns. For the spatial distribution of ζ values mapped onto the protein structures, refer to **Figure 4** in the main text.

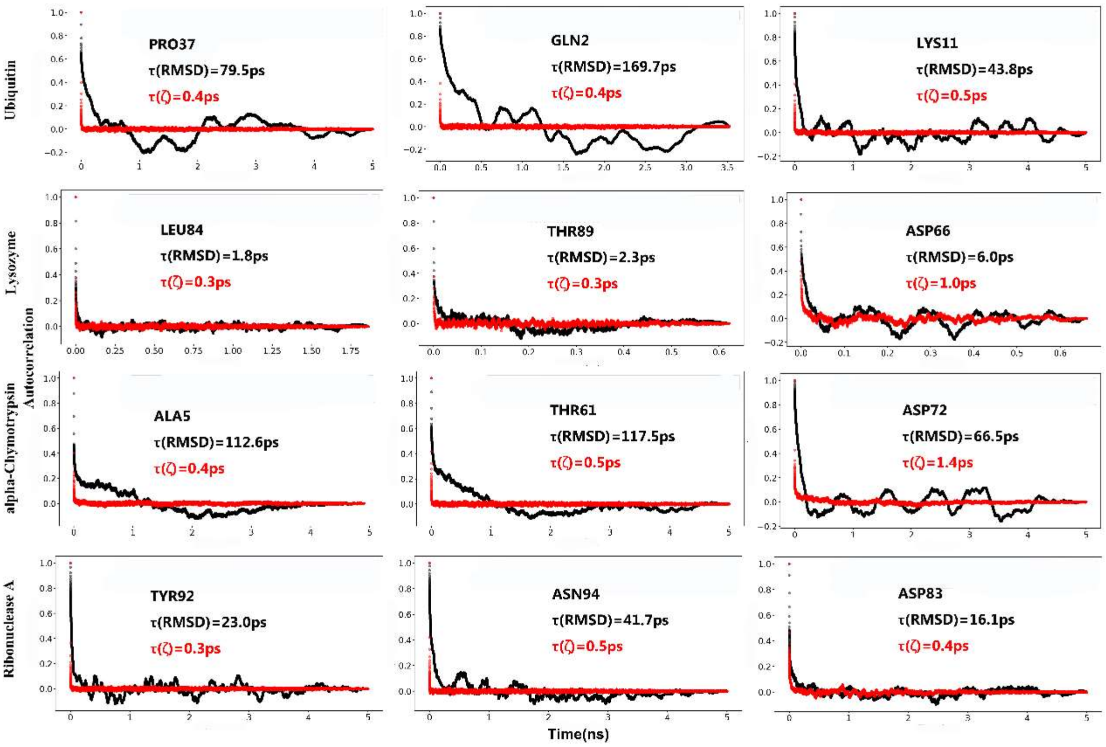

Figure S5. Scatter plots of the autocorrelation functions C(τ) for RMSD and ζ, along with their corresponding relaxation times (τ_ζ or τ_{RMSD}), for a subset of residues from the four proteins.

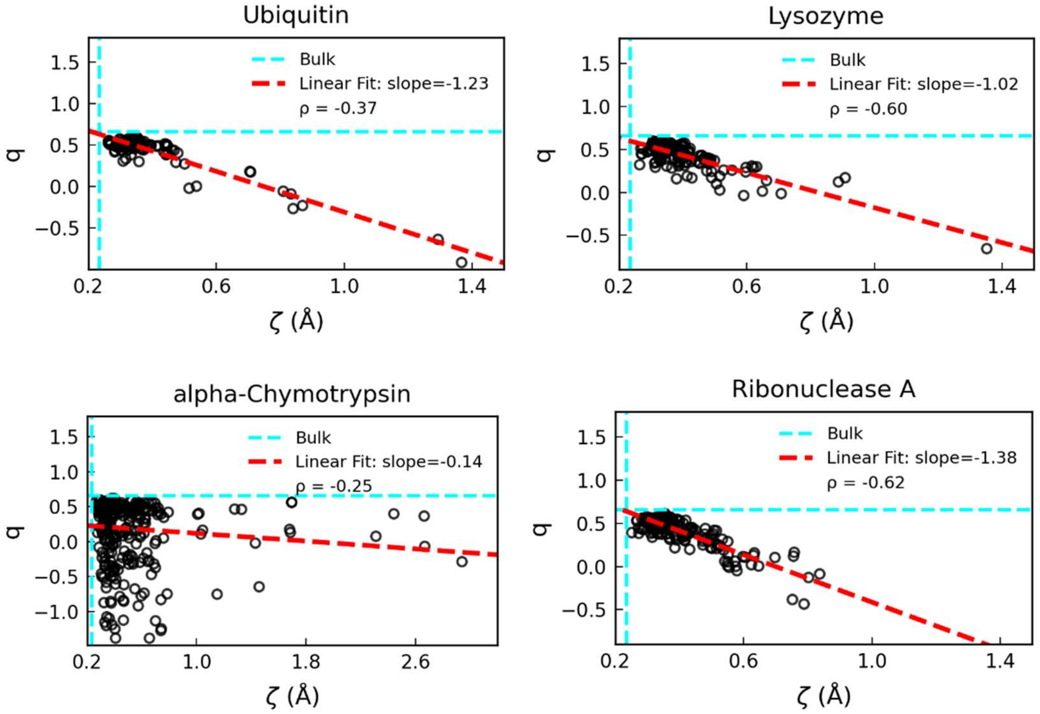

Figure S6. Correlations between the two structural order parameters (ζ and q) of hydration water for each surface residues from the four proteins (ubiquitin, lysozyme, α-chymotrypsin, and ribonuclease A) The correlation is quantified by spearman correlation coefficient (ρ) and a linear fitting (red dashed line) with slope and ρ shown on each subplot. Two blue dashed lines in the vertical and horizontal directions on each subplot denote the mean ζ (0.24 Å) and mean q value (0.66) of bulk water, respectively.
